# *CA8* Mutations Cause a Novel Syndrome Characterized by Ataxia and Mild Mental Retardation with Predisposition to Quadrupedal Gait

**DOI:** 10.1371/journal.pgen.1000487

**Published:** 2009-05-22

**Authors:** Seval Türkmen, Gao Guo, Masoud Garshasbi, Katrin Hoffmann, Amjad J. Alshalah, Claudia Mischung, Andreas Kuss, Nicholas Humphrey, Stefan Mundlos, Peter N. Robinson

**Affiliations:** 1 Institute for Medical Genetics, Charité-Universitätsmedizin Berlin, Berlin, Germany; 2 Max Planck Institute for Molecular Genetics, Berlin, Germany; 3 Genetics Research Center, University of Social Welfare and Rehabilitation Sciences, Tehran, Iran; 4 University of Babylon, Babylon, Iraq; 5 Centre for Philosophy of Natural and Social Science, London School of Economics, London, United Kingdom; 6 Berlin-Brandenburg Center for Regenerative Therapies, Berlin, Germany; Stanford University School of Medicine, United States of America

## Abstract

We describe a consanguineous Iraqi family in which affected siblings had mild mental retardation and congenital ataxia characterized by quadrupedal gait. Genome-wide linkage analysis identified a 5.8 Mb interval on chromosome 8q with shared homozygosity among the affected persons. Sequencing of genes contained in the interval revealed a homozygous mutation, S100P, in carbonic anhydrase related protein 8 (CA8), which is highly expressed in cerebellar Purkinje cells and influences inositol triphosphate (ITP) binding to its receptor ITPR1 on the endoplasmatic reticulum and thereby modulates calcium signaling. We demonstrate that the mutation S100P is associated with proteasome-mediated degradation, and thus presumably represents a null mutation comparable to the *Ca8* mutation underlying the previously described waddles mouse, which exhibits ataxia and appendicular dystonia. *CA8* thus represents the third locus that has been associated with quadrupedal gait in humans, in addition to the *VLDLR* locus and a locus at chromosome 17p. Our findings underline the importance of ITP-mediated signaling in cerebellar function and provide suggestive evidence that congenital ataxia paired with cerebral dysfunction may, together with unknown contextual factors during development, predispose to quadrupedal gait in humans.

## Author Summary

We identified a homozygous missense mutation (S100P) in the gene encoding carbonic anhydrase VIII in a consanguineous Iraqi family in which affected siblings had mild mental retardation and congenital ataxia characterized by quadrupedal gait. The affected persons walk on their hands and feet with their legs held straight with a “bear-like” gait. Our results show that the mutation S100P induces proteasome-mediated degradation with a severe reduction of the level of CA8 protein. The waddles (*wdl*) mouse, a spontaneous animal model with ataxia, was previously shown to harbor a 19-bp deletion in *Ca8* that leads to an almost complete lack of detectable Ca8 protein, resulting in abnormalities in cerebellar synaptic transmission. Therefore, we speculate that the reduction in CA8 protein concentration associated with the S100P mutation could result in similar pathophysiological effects. With the current report, alterations at three gene loci (CA8, VLDLR, and a yet-to-be discovered gene on chromosome 17p) have been reported to be associated with quadrupedal gait. It is unknown whether quadrupedal gait is related to specific molecular abnormalities or is an adaptive response to ataxia in some circumstances. However, we note that ataxia associated with mutations at all three loci is congenital and also associated with mental retardation, which is not generally a feature of other hereditary ataxias.

## Introduction

The hereditary ataxias comprise a diverse groups of disorders characterized by loss of balance and coordination. They are classified as autosomal recessive, autosomal dominant, X-linked, and mitochondrial, and are clinically and pathogenetically diverse [1],[2]. The identification of genes associated with individual forms of ataxia has gone a long way towards the identification of factors responsible for development, homeostasis, and function of the cerebellum, which is the organ primarily affected in most forms of ataxia.

Recently, a form of ataxia characterized by quadrupedal gait has been described in several families. A syndrome of nonprogressive cerebellar ataxia and mental retardation associated with inferior cerebellar hypoplasia and mild cerebral gyral simplification was initially identified in patients with a disorder termed “dysequilibrium syndrome” (MIM 224050). This disorder was found to be due to a 199 kb deletion on chromosome 9p24 encompassing all of the very low-density lipoprotein receptor gene (*VLDLR*) and part of the poorly characterized gene LOC401491 [3]. Homozygous premature-truncation-codon (PTC) mutations in *VLDLR* were subsequently found to be associated with severe ataxia, cerebellar hypoplasia, dysarthria, and severe mental retardation. Affected persons walked on all four extremities. Although the affected persons could stand upright and even walk bipedally, they preferred quadrupedal walking [4],[5]. An unrelated Iranian family with a PTC mutation in *VLDLR* was subsequently reported in which affected persons had mental retardation, strabismus, short stature, disturbed equilibrium, and walking disability, but no tendency towards quadrupedal gait [6]. A further unrelated family with cerebellar hypoplasia, mental retardation, and quadrupedal gait demonstrated linkage to a locus on chromosome 17p [7].

The observation that some, but not all, *VLDLR* mutation carriers walk on all four extremities raised the question of whether quadrupedal gait is a functional adaptation that can be seen in congenital ataxia syndromes depending on unknown internal or external influences. Although many mammals and other animals can stand or walk bipedally for shorter or longer periods of time, the manner in which humans do so is unique to our species. Our upright posture is the result of constant skeletomuscular adjustments of posture. In contrast, other primates make frequent use of “holds” during locomotion. In effect, we are always a second or two away from falling down. Human bipedalism and higher cognition are closely integrated. Especially running places high requirements on cognition, such as the need to incorporate information about uneven parts of the ground ahead. Nonhuman primates such as gorillas can only run for short distances, and human children do not develop adult competence in walking and running until about the age of seven years [8].

In the current work, we report on mutations in the *CA8* gene in a consanguineous family from Iraq. Affected family members displayed a syndrome of ataxia and mild mental retardation (AMMR) and ambulate on all four extremities (quadrupedal gait). In contrast to the previously reported cases associated with *VLDLR* mutations and with a locus on chromosome 17p, affected persons have only mild mental retardation. Including *CA8* as described in the present report, there are now three gene loci, mutations in which are associated with ataxia and cerebral defects, whereby some, but not all affected persons display quadrupedal gait. We therefore suggest that congenital ataxia together with cerebral deficits may, together with other, currently unknown external or internal factors, predispose to quadrupedal gait in humans.

## Results

We identified an Iraqi family with a syndrome characterized by ataxia and mild mental retardation (AMMR). The healthy parents are first cousins, and four of eight sibs are affected. The parents claimed that the affected persons never learned to crawl on their knees as most infants do, but ambulated from infancy on with their legs held straight with a “bear-like” gait. They also claimed that attempts to teach the children to walk on two legs with crutches or other supports failed. Our own observations confirm that four of the affected children as adults are predominantly quadrupedal (see Video S1). They walk with straight legs, placing weight on the palms of their hands. Although the affecteds are able to walk on two legs for several steps, they tend to tumble into a quadrupedal position quickly, complain of lack of balance and occasionally fall from a sitting position. Affected persons were noted to have mild mental retardation and dysarthric, slurred speech, but there were no other symptoms such as retinopathy or pyramidal signs. Table 1 provides an overview of the clinical features observed in the four affected siblings encoded using terms of the Human Phenotype Ontology [9].

**Table 1:** Clinical features of the affected persons of the family presented in this work and summary of features found related to mutations in *VLDLR* and at a locus on chromosome 17p.

HPO term	CA8	*VLDLR*	17p
	S100P	ns/fs	unknown
Cerebellar ataxia (HPO:0001253),	Q	Q/B	Q/B
Cerebellar ataxia associated with quadrupedal gait (HP:0009878)			
Dysarthria (HP:0001260)	+	+ (or no speech)	+
Mental retardation, mild (HP:0001256)	Mild	Moderate/Severe	Severe
Mental retardation, moderate (HP:0002342)			
Mental retardation, severe (HP:0001261)			
Tremor (HP:0001337)	+	+/−	+
Seizures (HP:0001250)	−	very rare	+/−
Strabismus (HP:0000486)	+/−	+	+
Cerebellar hypoplasia (HPO:0001321)	n/a	+	+
Cortical gyral simplification (HPO:0009879)	n/a	+	+
Corpus callosum hypoplasia (HPO:0002079)	n/a	−	+

The features are coded using terms from the Human Phenotype Ontology [9]. Abbreviations: Q: quadrupedal gait (HPO:0001253); B: cerebellar ataxia with bipedal gait (annotated as HPO:0001253). Q/B: some affecteds show quadrupedal gait, some bipedal; Mild: mild mental retardation (HP:0001256), Moderate: moderate mental retardation (HP:0002342); severe: severe mental retardation (HP:0001261). **+**: present, **−**: absent; **+/−**: present in some, absent in other affected persons. ns/fs: Information summarized for nonsense/frameshift mutations in *VLDLR* R257X [4], c2339delT [4],[5] and R448X [6]. Information for the 17p locus summarized from ref. [7]. n/a: information not available.

**Figure 1:**
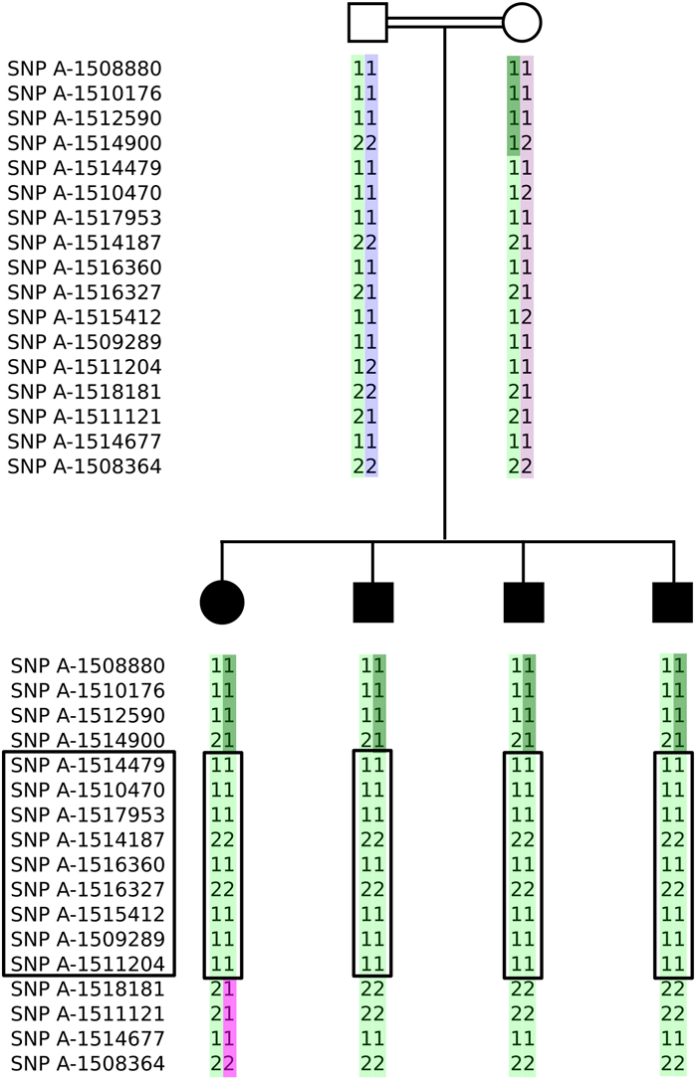
Abbreviated pedigree showing the inferred haplotypes of the parents and the four affected children. The SNPs A-1511204 and A-1509289 are homozygous in the mother and therefore noninformative for the maternally derived recombination in this region. Therefore, these markers were not fully informative with respect to the critical recombination on the maternally inherited haplotype in affected individual II-1.

We used a genome-wide linkage approach to identify the genetic basis of AMMR in this family. We established significant genetic linkage to a 6-centiMorgan interval on chromosome 8q12 with a lod score of 3.01 (Figure 1) This interval spans nucleotides 58,881,724–64,607,419 on chromosome 8 (March 2006, UCSC hg18 assembly) and contains 17 protein-coding genes, including an obvious candidate, carbonic anhydrase-related protein VIII (*CA8*), which is abundantly expressed in cerebellar Purkinje cells [10]. Carbonic anhydrases are a family of monomeric zinc metalloenzymes that catalyze the reversible hydration of CO_2_. However, CA8 lacks one of the three histidine residues required for binding to the zinc ion and thus has no catalytic carbonic anhydrase activity [11]. The waddles (*wdl*) mouse is a spontaneous animal model with ataxia and appendicular dystonia without morphological abnormalities in the central or peripheral nervous system. The *wdl* mouse was shown to harbor a 19-bp deletion in *Ca8* that leads to rapid degradation of mutant *Ca8* mRNA and to the almost complete lack of detectable Ca8 protein [12]. Although the morphology of the cerebellum appeared normal by confocal microscopy [12], subsequent investigations revealed abnormalities of parallel fiber-Purkinje cell synapses in the cerebellum together with defects in excitatory transmission [13].

**Figure 2:**
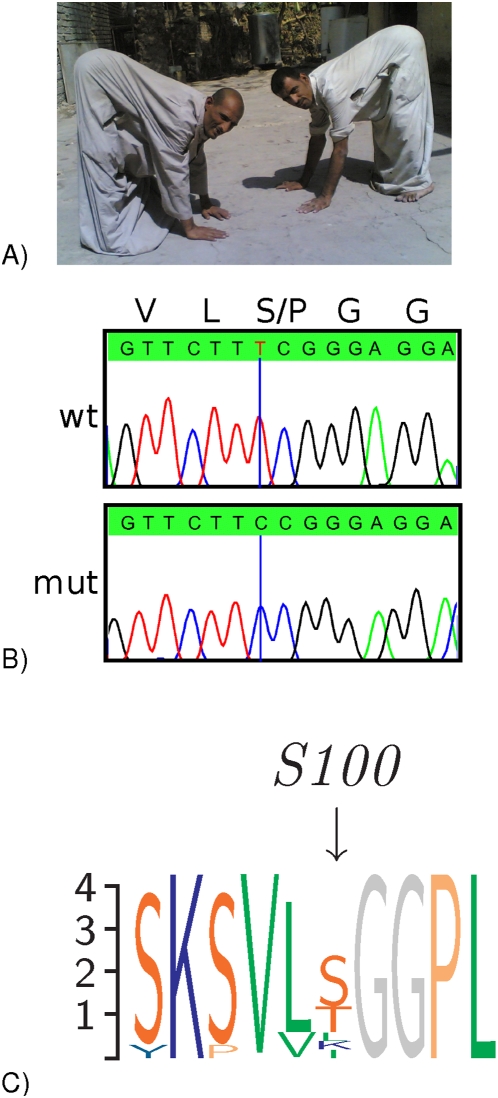
*CA8* Mutations in two families with ataxia and mild mental retardation. (A) Affected members of an Iraqi family displayed quadrupedal gait. (B) Sequence traces of the mutation c.298T>C (S100P) in *CA8* and a wildtype control (wt). (C) Sequence logo of an alignment of CA8 orthologs from 12 species from human to sea urchin showing the sequence affected by S100P. Position 100 does not show a high degree of conservation.

We therefore sequenced all 8 exons of *CA8* and identified the homozygous transition c.298T>C, predicted to lead to the substitution of a serine by a proline residue (S100P), in all affected individuals (Figure 2). c.298T>C segregated correctly with the disease in the family and was not found in 200 population-matched controls, thus making a previously undescribed polymorphism unlikely. In addition, we found no other nonsynonymous sequence changes in any of the other 16 genes in the mapping interval, including the *TTPA* gene, mutations in which are found in ataxia with vitamin E deficiency [14] (AVED).

**Figure 3:**
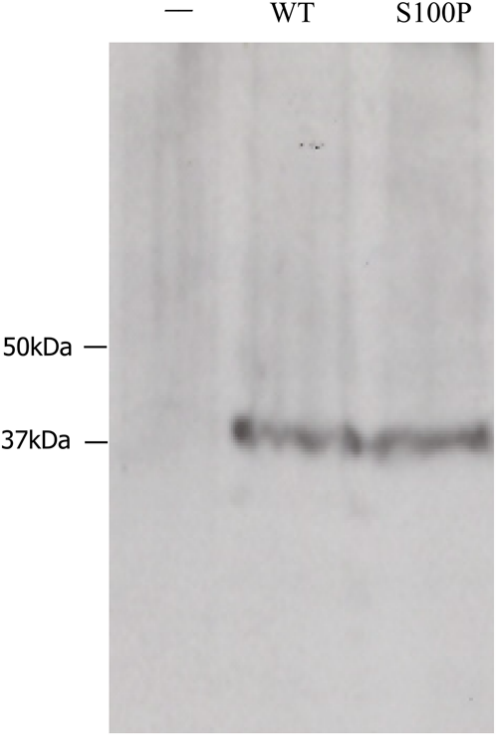
Blot overlay analysis of CA8-ITPR1 binding. A recombinant fibrillin-1 fragment rFib47^wt^ [32] was used as negative control (−).

The sole known biochemical function of CA8 is to inhibit inositol 1,4,5-triphosphate (IP_3_) binding to IP_3_ receptor 1 (ITPR1) [15]. ITPR1 plays a critical role in the modulation of intracellular calcium (Ca^2+^) signaling [16]. Like CA8, ITPR1 is also highly expressed in Purkinje cells. Mutation of *Ip3r1* underlies ataxia in mice and mutations in *ITPR1* have been identified in spinocerebellar ataxia 15 in humans [17]. Interestingly, mutant ataxin-3, which is the protein product of the gene mutated in spinocerebellar ataxia type 3 (SCA3), was shown to bind to ITPR1 and thereby cause a destabilization of neuronal calcium signaling [18]. These observations suggest that disturbances of ITPR1-mediated calcium signaling may be an important and common phenomenon in hereditary ataxias. We therefore investigated whether S100P affects CA8-ITPR1 binding using recombinant full-length CA8 and a region of ITPR1 containing the known CA8-binding domain [15]. No detectable difference was observed between wildtype and mutant CA8 constructs using blot-overlay assays (Figure 3). Therefore, since the *wdl* mouse displays a hypomorphic *Ca8* mutation [12], we hypothesized that S100P might affect protein stability.

**Figure 4:**
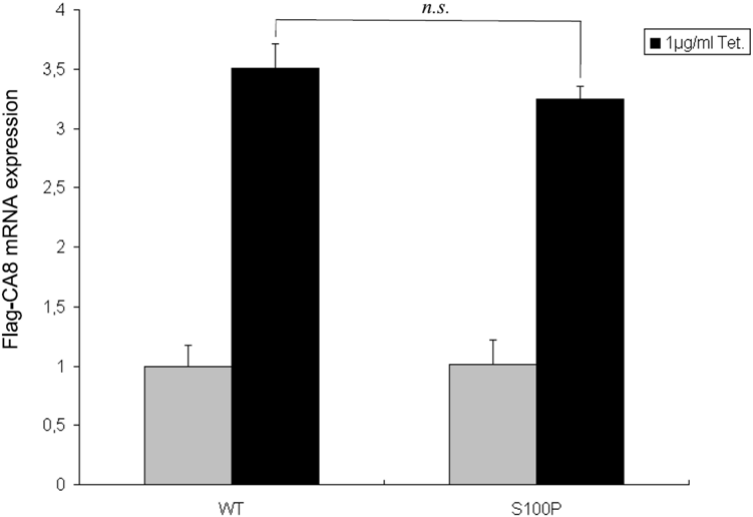
Relative expression levels of Flag-CA8 WT and S100P, tetracycline-inducible Flip-In T-REx-293 cells were grown in the absence or presence of 1 µg/ml tetracycline for 24 hours. GAPDH was used to normalize the individual expression levels (run in triplicate). There is no significant difference between the mRNA levels of mutant or wildtype *CA8*. *n.s.*: Non-significant.

**Figure 5:**
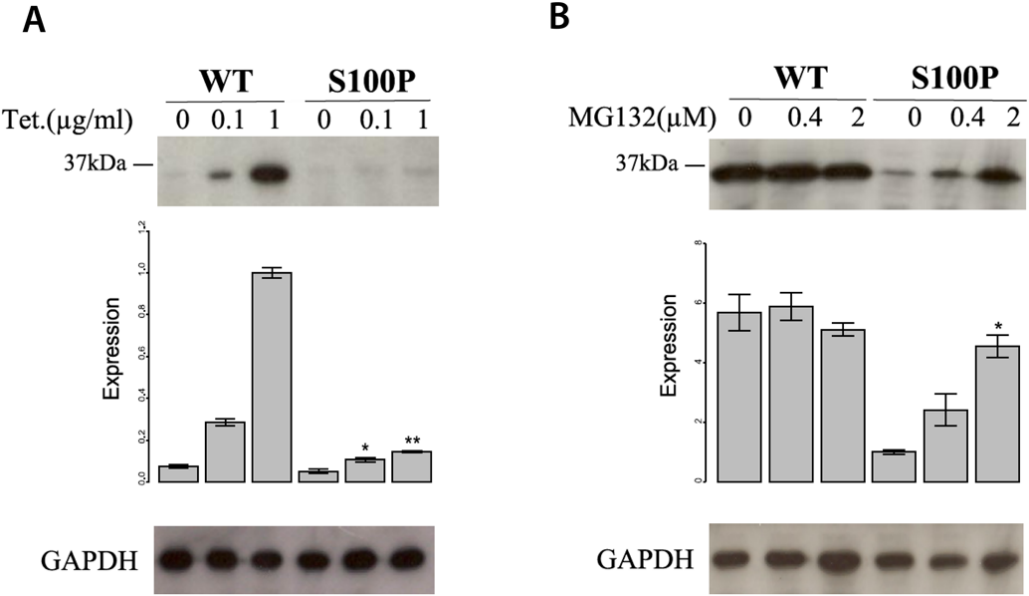
The mutation S100P reduces CA8 protein stability by means of proteasome-mediated CA8 degradation. (A) Reduction of CA8 protein concentration by S100P. Production of wildtype and mutant CA8 was induced by tetracycline. There was a strong reduction in the level of mutant CA8 protein compared to that of the wildtype at both 0.1, and 1.0 µg/ml tetracycline. *: 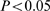, **: 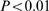, comparison between mutant and wildtype at the indicated tetracycline concentration by two-sided *t*-test. The expression is represented as signal intensity ratio between CA8/GAPDH, which was normalized to the WT level induced by 1 µg/ml tetracycline. Mean values±standard deviation (SD) in triplicate experiments are shown. (B) Rescue of mutant CA8 protein expression by proteasomal inhibition. Addition of the proteasome inhibitor MG132 lead to a dose-dependent rescue of CA8 concentration. *: 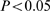, comparison between 0 µM MG132 and 0.4 µM or 2.0 µM MG132. Expression is shown as ratio relative to that of the mutant CA8 protein (S100P) without MG132. Mean values±SD in triplicate experiments are shown.

HEK cells were stably transfected with Flag-tagged wildtype and S100P mutant *CA8* constructs using a system that allows single-copy targeted integration of a tetracyclin-inducible construct. This allowed us to compare the stability of mutant and wildtype CA8 mRNA and protein at several different levels of expression. Following selection of stable clones, the expression of wildtype and mutant *CA8* was examined using quantitative realtime PCR, and the quantity of wildtype and mutant protein was examined by Western blots. Whereas mRNA levels for the mutant CA8 were not different from those of the wildtype construct (Figure 4), mutant CA8 protein was strongly reduced, being only barely detectable by Western blotting (Figure 5A). The level of mutant CA8 protein could be partially rescued using MG132, a proteasome inhibitor (Figure 5B). Taken together, these results indicate that S100P causes a reduction of protein stability owing to accelerated proteasomal degradation, which could be related to protein misfolding or other factors.

## Discussion

The cerebellum is a complex neurological structure, containing more than half of the brain's total number of neurons. Cerebellar networks show long-term synaptic plasticity, which indicates that experience-dependent adaptive and learning processes are a salient feature of cerebellar function. Most afferent information enters the cerebellum via climbing fibers (CF) and mossy fibers, which excite the Purkinje cells indirectly through the parallel fiber (PF) pathway. Binding of CA8 to ITPR1 inhibits IP_3_ binding to ITPR1 by reducing the affinity of the receptor for IP_3_ [15]. This is one of several factors that modulate the ability of ITPR1 to rapidly release calcium stores from the endoplasmatic reticulum [19]. Mice with mutations in either *Ip3r1* or *Ca8* do not display cerebellar atrophy, but rather both show neurophysiological defects [20],[21]. Modulation of intracellular calcium is important for a number of cerebellar functions such as long-term depression [22]. Therefore, we speculate that the consequences of a *CA8* mutation may involve improper modulation of the ITPR1 with resultant functional and/or developmental defects in the cerebellum.

S100P leads to a proteasome-mediated reduction in protein stability in our *in vitro* assay. Loss of protein stability is a common mechanism of missense mutations associated with human disease [23]. We suggest that it is plausible that the mutation leads to a reduction of the amount of CA8 in the cerebellum of the affected individuals, which might lead to a similar defect as that observed in the waddles mouse, in which Ca8 is nearly undetectable in cerebellum owing to a 19 bp deletion in the gene [12].

Mutations at three loci (*CA8*, *VLDLR* and the yet-to-be discovered gene at 17p) have now been found to be associated with quadrupedal locomotion in humans, although not in all affected individuals. The clinical picture of the disorders associated with mutations at the three loci is similar but shows important differences. The affected persons of the family described in this work showed a relatively mild degree of mental retardation. Seizures have not been observed in the family described in this study, but did represent a characteristic clinical feature of the family in whom linkage to a locus on chromosome 17p was reported [7]. Table 1 gives an overview of the clinical features encountered in the three forms of ataxia which have been observed with quadrupedal gait. Given the variable incidence of quadrupedalism in individuals with mutations in the same gene, we think it probable that contextual factors during development – either internal or external – contribute to this particular phenotypic outcome [24]. As one possibility, we note that ataxia associated with mutations at all three loci is congenital and also associated with mental retardation, which is not generally a feature of other hereditary ataxias, such as Joubert syndrome [25] or AVED [14]. Thus, perhaps it is only when congenital ataxia is coupled to a certain kind of malfunction of the cerebral cortex that individuals are likely to remain walking on all fours.

## Materials and Methods

Written informed consent was obtained from the subjects to publish details of their case and family history including the photograph and video documentation.

### Genome-Wide Linkage Analysis

The family was genotyped using the Affymetrix GeneChip® Human Mapping 10 K Array. The maximum expected lod score for a first cousin marriage with 4 affected children is approximately 3.01. The two-point and multipoint linkage analyses (Genehunter [26], Allegro [27] and Merlin [28]) were performed assuming a fully penetrant autosomal recessive trait with a disease frequency of 0.001 and no phenocopies. Analyses were performed using the easyLINKAGE and easyLINKAGE-Plus tools [29],[30].

### Recombinant Constructs

All PCRs were performed using High-Fidelity Taq Polymerase (Invitrogen). Two large amplicons comprising the full-length human *CA8* transcript and a region of *ITPR1* containing the known CA8-binding domain [15] (amino acids 1387–1647) of *ITPR1* were amplified by PCR from human fibroblast cDNA. A *CA8* amplicon corresponding to positions +91 to +1190 (numbering based on GenBank entry AY075022) was amplified using the primers 5′- tgcactcacactgcggttca-3′ (f) and 5′- aagggcattataggaccact-3′ (r). An *ITPR1* amplicon corresponding to positions +4361 to +5270 (numbering based on GenBank entry L38019) was amplified using the primers 5′-ctcatgtaccacatccactt-3 (f) and 5′-ccttaatgcagagcttctct-3′ (r).

We first produced CA8 and ITPR1 constructs for investigation of protein-protein binding. A recombinant CA8 construct corresponding to the complete coding region of human *CA8* was then amplified by nested PCR with the primers 5′-gtatGGATCCgatggcggacctgagcttca-3′ (f) and 5′- cttgCTCGAGctactgaaatgcagctctaa-3′ (r). Similarly, a recombinant *ITPR1* was amplified with the primers 5′- gtatGGATCCgaagaatgtctacacagaga-3′ (f) and 5′-cttgCTCGAGttatttcacatttccttctggcgt-3′ (r). The resulting fragments were subcloned into the plasmid pFastBac HT A (Invitrogen) using the *Bam*H1 and *Xho*1 restriction sites (capitalized in the primer sequences). pFastBac HT A adds a hexa-histidine tag and an rTEV protease cleavage site to the N-terminus of the expressed protein.

The c.298T>C (S100P) mutant CA8 construct was prepared by using the GeneTailor Site-Directed Mutagenesis kit (Invitrogen) using the primers 5′- tcaaaatcagttcttCcgggaggaccattgc-3′ (f) and 5′-aagaactgattttgacttcaggataacctga-3′ (r) according to the manufacturer's protocol.

Additional Flag-tagged CA8 constructs were produced for investigation of the influence of S100P on protein stability. PCR amplification of the full-length wild-type or S100P mutant CA8-pFastBac HT A constructs was used to subcloned to the *Bam*H1 and *Xho*1 sites of pcDNA3 (Invitrogen) using the primers 5′-gtatGGATCCatggcggacctgagcttca-3′ (f) and 5′-tgCTCGAGctacttgtcatcgtcgtccttgtagtcctgaaatgcagctct-3′ (r). The reverse primer introduces the Flag tag sequence DYKDDDDK to the C-terminus of the CA8 protein. pcDNA5/FRT/TO Flp-In constructs were then obtained by subcloning Flag-CA8(WT) and Flag-CA8(S100P) from pcDNA3.1 into the pcDNA5/FRT/TO vector using the *Bam*H1 and *Xho*1 restriction sites. All resulting recombinant vectors were verified by sequencing.

### Expression of Recombinant Human CA8 Protein and the CA8 Binding Domain of ITPR1

*Spodoptera frugiperda* (Sf9) cells were maintained in serum-free Insect-Xpress medium (Biowhittaker) supplemented with 5% fetal bovine serum, 10 U/ml penicillin, and 10 µg/ml streptomycin, and cultured at 27°C.

The production of the protein was performed according to the protocol of the Bac-to-Bac Baculovirus expression system (Invitrogen). Briefly, the recombinant pFastBac HT A vectors were transformed into *E. coli* DH10Bac cells (Invitrogen). The isolated positive recombinant bacmids were verified by PCR and used to transfect Sf9 insect cells for viral particle formation. Following three rounds of amplification of baculovirus, the virus titer was determined by plaque assay.

Small scale time-course expression experiments were conducted in 6 well plates to optimize the multiplicity of infection (MOI) and expression time. Bands corresponding to the size of recombinant protein were visualized on Western blots by detection with monoclonal anti-His6 antibody (Novagen). For large-scale production of protein, 5×10^8^ Sf9 cells were infected at an MOI of 1 plaque-forming unit (pfu)/cell with recombinant baculovirus. After about 48 hours cells were harvested and resuspended in 10 ml lysis buffer (10 mM Tris-HCl, 10 mM Imidazol, 25× EDTA free protease inhibitor cocktail (Sigma-Aldrich), 0.1 mM PMSF), sonication was performed on ice for 2×15 s at 50% maximum energy output and was cleared by centrifugation at 12,000 rpm for 30 min. Purification of the recombinant protein was performed as described before [31]. Concentration of the recombinant polypeptide was determined with the BCA protein assay (Pierce) according to the manufacturer's instruction.

### Blot Overlay Assay

For the blot overlay assay, recombinant wildtype and S100P mutant CA8 were separated by sodium dodecyl sulfate-12% polyacrylamide gel electrophoresis (SDS-PAGE) and then transferred onto a polyvinylidene difluoride (PVDF) membrane (Immobilon-P transfer membrane, Millipore). A recombinant fibrillin-1 fragment [32] was used as a control. Non-specific binding sites were blocked with dilution buffer (ProFound Far-Western Biotinylated Protein∶Protein Interaction Kit, Pierce), according to the manufacturer's instructions, and incubation was performed for 2 h at room temperature with 2 µg/ml of the biotinylated ITPR1 fragment in dilution buffer and was followed by washing the PVDF membrane three times with PBS including 0.025% Tween 20. For detection, incubation was performed with streptavidin-horseradish peroxidase conjugate for 3 h at room temperature. Membranes were washed six times in 1× PBS including 0.025% Tween 20. Membranes were then incubated in the UnBlot substrate working solution (ProFound Far-Western Biotinylated Protein∶Protein Interaction Kit, Pierce). Hyperfilm ECL chemiluminescence films (Amersham Pharmacia) were exposed according to the manufacturer's instructions.

### Generation of Stable Flp-In T-REx 293 Cells Expressing Flag-CA8(WT) and Flag-CA8(S100P)

To generate stable tetracycline-inducible Flp-In T-REx cells, 0.5 µg pcDNA/FRT/TO encoding Flag-CA8(WT) or Flag-CA8(S100P)] or empty pcDNA/FRT/TO and 1 µg pOG44 were cotransfected into Flp-In T-REx 293 cells (1×10^6^) using Lipofectamine 2000 (Invitrogen) according to the manufacturer's directions. 48 hours after transfection cells were reseeded at less than 25% confluence, after 4 hours, the medium was changed to medium supplemented with 100 µg/ml hygromycin B (Invitrogen) and 15 µg/ml blasticidin. After two weeks, hygromycin-resistant colonies were picked and sub-cultured. Selection of positive colonies was performed by immunoblotting as described below.

### Detection of mRNA by Quantitative RT-PCR

Tetracycline inducible Flip-In T-REx 293 cells were grown in the absence or presence of 1 µg/ml tetracycline for 24 hours, total RNA of those cells were extracted with NucleoSpin RNA II (Macherey-Nagel, Duren, Germany) according to the manufacturer's protocol. 1 µg RNA from cells was reverse-transcribed using the Superscript First Strand synthesis kit (Invitrogen). Quantitative RT-PCR was performed on an ABI Prism 7500 Sequence Detection System using SYBR-Green PCR Master Mix at 50°C for 2 min, 95°C for 10 min, then 40 cycles at 95°C for 15 s, and at 60°C for 1 min. The forward primer for *CA8* is complementary to a sequence at the C-terminal region of *CA8*, 5′-cttgtggaaggctgtgatgg-3′, and the reverse primer is complementary to a sequence of the Flag tag, 5′-ttgtcatcgtcgtccttgtag-3′. The primer pair for amplification of *GAPDH* was: 5′-ctcaacgaccactttgtcaagctca-3′, 5′-ggtcttactccttggaggccatgtg-3′.

### Immunoblotting to Detect Flag-CA8 Proteins

To detect the expression of CA8-Flag WT and S100P protein, tetracycline-inducible Flip-in T-REx cells seeded in 24 well plate (2×10^5^) were induced by 0.1 µg/ml or 1 µg/ml tetracycline (Sigma-Aldrich). To inhibit the proteasome, cells were incubated in proteasome inhibitor MG132 (Sigma-Aldrich) at different concentrations (0.4 µM, 2.0 µM) in the presence of 1 µg/ml tetracycline. After 24 hours cells were washed twice with PBS and lysed with 100 µl of 1× SDS-sample buffer (50 mM Tris-HCL pH 6.8, 10% glycerol, 2% SDS, 1% 2-mercaptoethanol, 0.02% Bromphenol blue). After brief sonication on ice, 10 µl aliquots were separated on 12% SDS-gel and electrophoretically transferred to a PVDF membrane. Immunodetection was performed by using mouse monoclonal anti-Flag (1∶2000, Sigma-Aldrich) as primary antibody, followed by subsequent incubation with HRP (horseradish peroxidase)-conjugated rabbit anti-mouse secondary antibody (1∶2000, Calbiochem). Parallel Western blots were probed with a mouse anti-GAPDH antibody (1∶5000, Abcam) as a loading control. Protein expression was quantified using the ImageJ program [33].

### CA8 Sequence Logos

A CA8 protein multiple alignment from Human (*Homo sapiens*; NP_004047), dog (*Canis familiaris*; XP_544094), cow (*Bos taurus*; NP_001077159), horse (*Equus caballus*; XP_001496523), mouse (*Mus musculus*; NP_031618), rat (*Rattus norvegicus*; NP_001009662), opossum (*Monodelphis domestica*; XP_001368351), chicken (*Gallus gallus*; XP_419221), frog (*Xenopus (Silurana) tropicalis*; NP_001011213), trout (*Oncorhynchus mykiss*; NP_001118116), zebrafish (*Danio rerio*; NP_001017571), and sea urchin (*Strongylocentrotus purpuratus*; XP_795365) was prepared with ClustalX [34] and sequence logos were prepared using texshade [35].

## Supporting Information

Video S1 Film demonstrating quadrupedal gait of one of the families described in this manuscript.
(1.94 MB WMV)
